# The Three Receptor Tyrosine Kinases c-KIT, VEGFR2 and PDGFRα, Closely Spaced at 4q12, Show Increased Protein Expression in Triple-Negative Breast Cancer

**DOI:** 10.1371/journal.pone.0102176

**Published:** 2014-07-15

**Authors:** Sara Jansson, Pär-Ola Bendahl, Dorthe Aamand Grabau, Anna-Karin Falck, Mårten Fernö, Kristina Aaltonen, Lisa Rydén

**Affiliations:** 1 Division of Oncology and Pathology, Department of Clinical Sciences Lund, Lund University, Lund, Sweden; 2 Department of Surgery, Hospital of Helsingborg, Helsingborg, Sweden; 3 Division of Surgery, Department of Clinical Sciences Lund, Skåne University Hospital, Lund, Sweden; University of Parma, Italy

## Abstract

**Background:**

Triple-negative breast cancer (TNBC) is a heterogeneous subgroup of breast cancer with poor prognosis and no targeted therapy available. Receptor tyrosine kinases (RTKs) are emerging targets in anticancer therapy and many RTK-inhibiting drugs are currently being developed. The aim of this study was to elucidate if there is a correlation between the protein expression of three RTKs c-KIT, VEGFR2 and PDGFRα, their gene copy number, and prognosis in TNBC compared to non-TNBC.

**Methods:**

Tumor tissue samples from patients diagnosed with primary breast cancer were stained with immunohistochemistry (IHC) for protein assessment, and with fluorescence *in situ* hybridization (FISH) for gene copy number determination. Breast cancer mortality (BCM), measured from the date of surgery to death, was used as endpoint.

**Results:**

The cohort included 464 patients, out of which 34 (7.3%) had a TNBC. High expression of the three RTKs was more common in TNBC compared to non-TNBC: c-KIT 49% vs. 10% (*P*<0.001), PDGFRα 32% vs. 19% (*P* = 0.07) and VEGFR2 32% vs. 6% (*P*<0.001). The odds ratio (OR) of c-KIT, VEGFR2 and PDGFRα positivity, adjusted for tumor characteristics, was 6.8, 3.6 and 1.3 times higher for TNBC than for non-TNBC. 73.5% of the TNBC had high expression of at least one of the three investigated receptors, compared to 30.0% of the non-TNBC (*P*<0.001). Survival analysis showed no significant difference in BCM for TNBC patients with high vs. low c-KIT, PDGFRα or VEGFR2 protein expression. 193 (42%) tumors were evaluated with FISH. No correlation was seen between increased gene copy number and TNBC, or between increased gene copy number and high protein expression of the RTK.

**Conclusion:**

c-KIT, VEGFR2 and PDGFRα show higher protein expression in TNBC compared to non-TNBC. Further investigation clarifying the importance of these RTKs in TNBC is encouraged, as they are possible targets for anticancer therapy.

## Introduction

Breast cancer is a complex heterogeneous disease and it can be classified into several distinctive subgroups based on gene expression profiles [1]–[3]. This classification gives important information about prognosis and cellular molecular aberrations that could serve as targets for novel medical therapy. In clinical practice, immunohistochemical translations of the results of gene expression profiles are being used to allocate the patients to the different subgroups [4]–[6]. Guidelines for classification in the clinical setting have been proposed by St Gallen and are based on IHC analysis of the estrogen receptor (ER), the progesterone receptor (PR) and Ki67, and ISH-analysis of the human epidermal growth factor receptor 2 (HER2). By these analyses breast cancers are classified as luminal A (ER+ and/or PR+, Ki67 low and HER2−), luminal B HER2− (ER+ and/or PR+, Ki67 high and HER2−), luminal B HER2+ (ER+ and/or PR+, any Ki67 and HER2+), HER2-type (ER−, PR− and HER2+) and triple-negative (ER−, PR− and HER2−) [6].

The triple-negative breast cancer (TNBC) subgroup constitute approximately 12–17% of female breast cancers [7] and is associated with a particularly poor prognosis. Patients diagnosed with TNBC are often younger, have tumors with a higher histologic grade and are more frequently BRCA1 mutation carriers than those within the other breast cancer subgroups. They also tend to present with larger tumors at diagnosis [8], [9].

The TNBC group comprises a greater diversity of tumors than the other breast cancer subgroups and it has been proposed that the TNBC group should be further subdivided based on the expression of basal breast cell markers (CK5/6 and/or EGFR) [5], [10]–[13]. Another study further consolidating the heterogeneity of TNBC was performed by Lehnmann *et al.* where gene expression profiling analysis was performed on a set of 587 TNBC tumors identifying 6 stable TNBC subtypes [14].

At present, the mainstay of adjuvant systemic treatment for TNBC is chemotherapy [7], [9], compared to the other breast cancer subgroups where ER and HER2 provide targets for therapy. Potential targets in the TNBC group are currently being investigated [15] and receptor tyrosine kinases (RTKs) are emerging as such [16]. A RTK is a transmembrane receptor protein that upon binding of its ligand initiate an intracellular signal cascade ultimately leading to changes in the cell's gene expression and phenotype [17]. The best example of a successful RTK-inhibitor is imatinib that inhibits both c-KIT and platelet-derived growth factor receptor alpha (PDGFRα) and it is currently used for treating, amongst others, gastrointestinal stromal tumors (GISTs) and chronic myeloid leukemia (CML) [16]. Two other examples are sunitinib and sorafenib, which are multi-tyrosine kinase inhibitors [18], [19].

In this study, gene copy number and protein expression were evaluated for three RTKs as potential breast cancer drug targets: c-KIT, vascular endothelial growth factor receptor-2 (VEGFR2) and PDGFRα. The genes *c-KIT*, *VEGFR2* and *PDGFRα* are all adjacently located at the 4q12 chromosomal segment and their involvement in the cancer process have been investigated in various malignancies, such as for example gliomas [20]–[22], malignant peripheral nerve sheath tumors [23] and GISTs [24]. However, to date, their role in breast cancer remains largely unknown. High expression of c-KIT and VEGFR2 has previously been correlated to basal-like breast cancer (BLBC) and TNBC [25]–[28]. Overexpression of PDGFRα in breast cancer has been found to be associated with tumor progression [29] and to be involved in the metastasis process [30]. PDGFRα has recently been associated with basal B like cell lines [31], but to our knowledge, the expression of PDGFRα has not been correlated to a breast cancer subgroup in a clinical cohort before.

Gain-of-function mutations in *c-KIT* leading to pathologic activation are seen in several neoplasms, such as GISTs and acute myeloid leukemia [32]. A previous study has shown that increased gene copy number of *c-KIT* and *VEGFR2* in primary breast cancer is related to an aggressive phenotype and impaired prognosis [27].

The aim of this study was to analyze protein expression and gene copy number for *c-KIT*, *VEGFR2* and *PDGFRα* in order to elucidate if there is a correlation between the copy number of these genes, their protein expression, and the prognosis of breast cancer in the TNBC subgroup compared to non-TNBC.

## Materials and Methods

### Patients

The patient cohort used in this study was originally assembled for an observational prospective study with the aim of evaluating the presence and prognostic value of disseminated tumor cells in bone marrow. The study was approved by the ethics committee at Lund University, and all the included patients gave a written informed consent (LU699-09, LU75-02). Further information about the patient cohort has been published elsewhere [33], [34].

**Figure 1:**
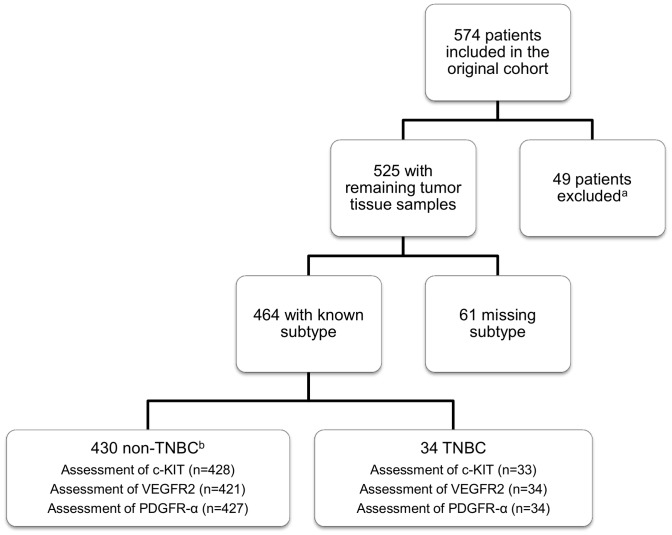
Flow-chart of the patient cohort included in this study. ^a^These patients were excluded because they did not meet the inclusion criteria (e. g. patients with local breast cancer recurrence, bilateral breast cancer, no breast cancer or too few cancer cells in tissue samples). ^b^TNBC = Triple-negative breast cancer.

In summary, patients diagnosed with primary breast cancer in the South Swedish Health Care Region between June 1999 and May 2003, were included in the original cohort. The patients were treated surgically with either mastectomy or breast-conserving therapy based on pre-operatively identified characteristics and staging. Axillary lymph node dissection was performed on patients with lymphatic metastatic spread diagnosed either before surgery or following sentinel node biopsy. Patients were recommended adjuvant therapy according to clinical standards following Regional Guidelines. Data on breast cancer related death was retrieved from the Swedish Register of Causes of Death (Central Statistics Office). The median follow-up time for patients alive and without any breast-cancer related event was 61 months. Detailed information on routine prognostic factors, St Gallen molecular subtype and clinical follow-up data were assembled for all patients as described in Falck *et al.* [34] In total, 464 patients with known breast cancer subtype and remaining evaluable tumor tissue samples were included in the present study (Figure 1).

### Tissue microarray

Formalin-fixed, paraffin embedded tumor tissue samples were retrieved from the Department of Pathology in Lund and Helsingborg, Sweden. Tissue micro arrays (TMAs) were constructed by extracting tissue core biopsies 1.0 mm in diameter from representative areas of invasive breast cancer using a tissue array machine (TMArrayer Pathology Devices, INC.). Two core biopsies were taken from each patient tumor sample. Biopsies were mounted into a recipient block and stored dark at room temperature until glass slide transfer and staining.

### Immunohistochemistry (IHC)

Sections between 3 and 4 µm thick were taken from each TMA, transferred to glass slides (Menzel Super frost plus, Thermo Scientific, Germany), dried at room temperature and then baked in a heat chamber for two hours at 60°C. After deparaffinisation and antigen retrieval, staining was performed using an Autostainer *Plus* (Dako Denmark A/S, Glostrup, Denmark). The following antibodies and dilutions were used: c-KIT (#A4502, Dako Denmark A/S, Glostrup, Denmark, diluted 1∶400), PDGFRα (#3164 Cell Signaling Technology, Inc., Danvers, MA, USA diluted 1∶100) and VEGFR2 (#2479 Cell Signaling Technology, Inc., Danvers, MA, USA diluted 1∶100). To amplify the signal of the primary PDGFRα antibody, a Rabbit Link K8009 (Dako Denmark A/S, Glostrup, Denmark) was used. A visualization kit K801021-2 (Dako Denmark A/S, Glostrup, Denmark) was used for all stainings, and all slides were counterstained with Mayer's Haematoxylin applied for two minutes.

Two investigators evaluated the IHC staining independently, SJ and DG for c-KIT, and SJ and KA for PDGFRα and VEGFR2. Stainings were evaluated for intensity 0–3 (0 = negative, 1 = weak, 2 = intermediate and 3 = strong) and percentage of stained tumor cells. Only invasive tumor cells were assessed and only TMA core biopsies with >100 tumor cells were included. Samples with differences in assessment between the two investigators were re-evaluated and a consensus decision taken. The highest value of two cores was used in the statistical analysis.

A TMA core biopsy was considered c-KIT positive whenever ≥1% of the cancer cells were stained, according to common practice [25]. No standard IHC assay protocols are available for VEGFR2 and PDGFRα, and assessments were based on previously published histoscore protocols. For VEGFR2, the percentages of stained cancer cells were grouped in 4 groups, (<5% = 0, 5–33% = 1, 34–66% = 2, 67–100% = 3). A score was calculated multiplying the fraction (0–3) with the intensity (0–3) resulting in a product between 0 and 9. A tumor tissue sample was considered VEGFR2 positive if the final score was >6 [35]. For PDGFRα, the percentages of stained cancer cells were grouped in 5 groups, (0% = 0, 1–9% = 1, 10–50% = 2, 51–80% = 3, 81–100% = 4). A score was calculated multiplying the fraction (0–4) with the intensity (0–3) resulting in a product between 0 and 12. A tumor tissue sample was considered PDGFRα positive if the final score was ≥5 [36].

### Fluorescence *in situ* hybridization (FISH)

A custom-made Poseidon quadruple-color DNA probe was ordered from Kreatech (Kreatech, Amsterdam, Netherlands). It was composed of a probe mix to detect *c-KIT* (labeled with PlatinumBright495, green), *VEGFR2* (labeled with PlatinumBright550, gold), *PDGFRα* (labeled PlatinumBright590, red) and the control region SE4 (labeled with PlatinumBright415, blue).

With some minor adjustments, staining was performed according to the manufacturer's manual using chemicals from Tissue Digestion Pretreatment Kit I (Kreatech, Amsterdam, Netherlands). TMA slides were baked for 2 hours in 80°C. They were deparaffinised in xylene for 2×7 min, and rehydrated 3 min each in absolute (ABS), 85% and 70% ethanol. The slides were treated for 15 min in Pretreatment A (97–98°C) and rinsed for 2×2 min in distilled water (dH_2_O). Pepsin was added to the slides for 40 min and the slides were rinsed in dH_2_O for 1 min. The slides were put in 2×SSC with pH 7.0 for 5 min, and dehydrated in 70%, 85% and ABS for 1 min each. After drying, 20 µl of Poseidon quadruple-color DNA probe was added and a cover glass was mounted on each TMA slide and sealed with rubber glue. The slides were put on a hot plate (80°C) for 5 min for co-denaturation and incubated in a humid dark chamber to hybridize overnight at 37°C. The next day, rubber glue and cover glass were removed; the TMA slides were dipped in Washbuffer II and rinsed for 2 min in Washbuffer I (73°C). Finally the slides were left in Washbuffer II for 1 min, followed by dehydration in 70%, 85% and ABS for 1 min each. When air-dried, 22 µl of DAPI counterstain (concentration 0.05 µg/ml) and cover glass were added.

Only one of the two core biopsies from each patient was examined. 30 invasive cancer cells were evaluated in each sample. The number of gene copies and of chromosome 4 control regions per cell was counted. A gain was considered when a cell contained ≥4 gene copies. If the ratio between gene copies and chromosome 4 control regions was >2 the cell was considered to have an amplification. Any tissue sample containing ≥5 cells with gains and/or amplifications was considered FISH positive. The cut-off point for FISH positivity was chosen based on a comparison of cut-off points used in recent similar studies [20], [21], [27], [37]. Since in this study TMAs of formalin-fixed tissue were used, we used the cut-off point that was closest to the one used by Joensuu *et al.* [20] who used the same type of tissue material.

### Statistical analysis

Breast cancer mortality (BCM) was chosen as endpoint in this study. Survival data was retrieved from the Swedish Register of Causes of Death (Central Statistics Office) and registered events until 31 of December 2010 were recorded.

To evaluate differences in the distribution of clinical data and tumor characteristics between the non-TNBC group and the TNBC group a χ^2^ test was used. Binary logistic regression analysis was performed to quantify the effect of each RTK, with and without adjustment for other tumor characteristics.

The effect on survival for high protein expression and increased gene copy number of c-KIT, VEGFR2 and PDGFRα, was described in terms of BCM using cumulative incidence curves. *P*-values were calculated using the log-rank test and Hazard ratios (HR) using Cox regression. *P*-values<0.05 were considered statistically significant. The statistical calculations were performed using SPSS Version 21.0 (SPSS, Chicago, IL) and graphs were drawn in Stata Version 12.1 (StataCorp LP, College Station, TX).

## Results

### Patient cohort and TNBC patient characteristics

34 (7.3%) of the included 464 tumors were diagnosed as triple-negative (Figure 1). The TNBC presented with larger tumors at diagnosis (*P* = 0.07), higher Nottingham histological grade (NHG) (*P*<0.001) and the Ki67 index (*P*<0.001), compared to the non-TNBC (Table 1). Furthermore, the TNBC patients were younger (*P* = 0.008) and had a 2.7-fold higher BC mortality (95% CI 1.3–5.7, *P* = 0.009) than those carrying a non-TNBC.

**Table 1:** Patient and tumor characteristics in relation to triple-negative breast cancer (TNBC).

Characteristics^a^	All patients N (%)	non-TNBC N (%)	TNBC N^b^ (%)	*P*-value
	N = 464	N = 430	N = 34	
Age				
Median (range)	58 (26–88)	58 (26–88)	52 (29–86)	
<50	95 (20)	82 (19)	13 (38)	0.01^c^
≥50	369 (80)	348 (81)	21 (62)	
Histopathologic type				
Ductal	329 (71)	301 (70)	28 (82)	<0.001^c^
Lobular	85 (18)	85 (20)	0	
Tubular	18 (4)	18 (4)	0	
Medullary	11 (2)	6 (1)	5 (15)	
Mainly DCIS	12 (3)	12 (3)	0	
Other	9 (2)	8 (2)	1 (3)	
Unknown	0	0	0	
Tumor size				
≤20 mm	310 (67)	292 (68)	18 (53)	0.09^c^
>20 mm	153 (33)	137 (32)	16 (47)	
Unknown	1	1	0	
Node status				
N+	190 (42)	175 (42)	15 (44)	0.86^c^
N0	264 (58)	245 (58)	19 (56)	
Unknown	10	10	0	
NHG				
1	101 (22)	101 (24)	0	<0.001^e^
2	243 (53)	235 (55)	8 (24)	
3	115 (25)	89 (21)	26 (77)	
Unknown	5	5	0	
Ki67				
High (>20%)	157 (34)	131 (31)	26 (77)	<0.001^c^
Low (≤20%)	307 (66)	299 (70)	8 (24)	
Unknown	0	0	0	

a DCIS = ductal cancer *in situ*; N0 = node negative; N+ = node positive; NHG = Nottingham histological grade.

b Percentages are shown despite the small number of patients in this group (N<50).

c *P*-value from Fisher's Exact Test.

e *P*-value from Linear-by-Linear Association test.

32 (94%) of the 34 TNBCs had a Core Basal phenotype (defined as being negative for ER, PR and HER2, and positive for CK5/6 and/or EGFR).

### Protein expression in TNBC compared to non-TNBC

**Figure 2:**
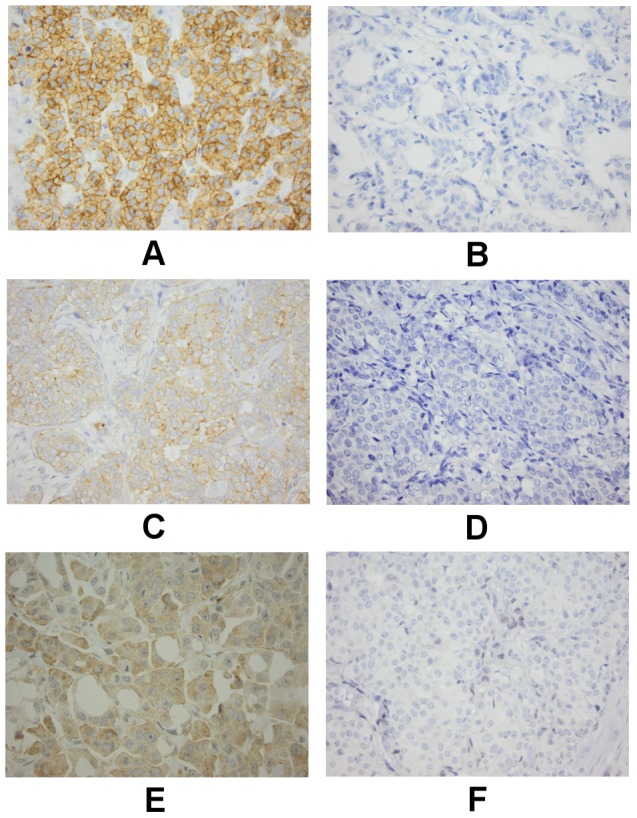
The left panels show examples of positive immunohistochemical staining with strong intensity for c-KIT (A), VEGFR2 (C) and PDGFRα (E). Negative controls are shown to the right, c-KIT (B), VEGFR2 (D) and PDGFRα (F). Original magnification ×40.

Examples of IHC staining are shown in Figure 2A–2F and the results are summarized in Table 2. Test for correlations between the expressions of the three proteins are shown in Table S1. Table S2 a, b and c presents the distribution of the tumors between the percentage groups (c-KIT) and the IHC scores (VEGFR2 and PDGFRα) for TNBC and non-TNBC. Cut off points has been marked by a separating space in each table to demonstrate the rationale behind the cut off points. Significantly higher expression of c-KIT and VEGFR2 was found in TNBC compared to non-TNBC tumors (*P*<0.001) and PDGFRα showed the same tendency (*P* = 0.07).

**Table 2:** Protein expression and gene copy number of c-KIT, VEGFR2 and PDGFRα in relation to triple-negative breast cancer (TNBC).

IHC^a^ and FISH^a^ marker	All patients N (%)	Luminal A N (%)	Luminal B HER2− N (%)	Luminal B HER2+ N (%)	HER2+ N (%)	TNBC N (%)	*P*-value for St Gallen subgroup	*P*-value TNBC *vs* non-TNBC
c-KIT IHC								
Positive	57 (12)	20 (8)	13 (16)	8 (11)	0 (0)	16 (49)	<0.001^b^	<0.001^b^
Negative	404 (88)	233 (92)	67 (84)	66 (89)	17 (100)	17 (52)		
Unknown	3	2	0	0	0	1		
VEGFR2 IHC								
Positive	43 (10)	18 (7)	6 (8)	7 (10)	1 (6)	11 (32)	<0.001^b^	<0.001^b^
Negative	412 (90)	230 (93)	73 (92)	67 (91)	15 (94)	23 (68)		
Unknown	9	7	1	0	1	0		
PDGFRα IHC								
Positive	94 (20)	38 (15)	24 (30)	15 (20)	3 (18)	11 (32)	0.02^b^	0.07^b^
Negative	367 (80)	215 (85)	55 (70)	59 (80)	14 (82)	23 (68)		
Unknown	3	2	1	0	0	0		
*c-KIT* FISH								
Positive	21 (11)	11 (11)	4 (10)	3 (10)	1 (14)	2 (11)	1^b^	1^b^
Negative	172 (89)	86 (89)	36 (90)	27 (90)	6 (86)	16 (89)		
Unknown	271	158	40	44	10	16		
*VEGFR2* FISH								
Positive	22 (11)	10 (10)	5 (13)	4 (13)	1 (14)	2 (11)	1^b^	1^b^
Negative	171 (89)	87 (90)	35 (88)	26 (87)	6 (86)	16 (89)		
Unknown	271	158	40	44	10	16		
*PDGFRα* FISH								
Positive	24 (12)	12 (12)	5 (13)	4 (13)	1 (14)	2 (11)	1^b^	1^b^
Negative	169 (88)	85 (88)	35 (88)	26 (87)	6 (86)	16 (89)		
Unknown	271	158	40	44	10	16		

a IHC = immunohistochemistry. FISH = fluorescence *in situ* hybridization.

b *P*-value from Fisher's Exact Test.

Binary logistic regression showed that the unadjusted odds ratio (OR) of c-KIT positivity was 8.9 times higher for TNBC cases than for non-TNBC cases (95% CI 4.2–19, *P*<0.001). The corresponding unadjusted OR of VEGFR2 positivity was 5.8 (95% CI 2.6–13, *P*<0.001), and of PDGFRα positivity 2.0 (95% CI 0.9–4.2, *P* = 0.08). The OR of c-KIT positivity, adjusted for histopathological type, grade, tumor size >20 mm, and lymph node engagement, was 6.8 times higher for TNBC cases than for non-TNBC cases (95% CI 2.9–16, *P*<0.001) and the corresponding adjusted OR of VEGFR2 positivity was 3.6 (95% CI 1.4–9.3, *P* = 0.007), and of PDGFRα positivity 1.3 (95% CI 0.6–3.1, *P* = 0.5).

To further investigate the connection between these three RTKs and TNBC we also analyzed how many tumors were positive for at least one, and at least two, of the three receptors. 25 (73.5%) of the 34 TNBC tumors had a high expression of at least one of the three receptors compared to 129 (30.0%) of the 430 non-TNBC (*P*<0.001). 12 (35.3%) of the 34 TNBC tumors showed a high expression of at least two of the three receptors compared to 25 (5.8%) of the 429 non-TNBC (*P*<0.001). Only three tumors in total were positive for all three RTKs, one of them was a TNBC. OR for a TNBC patient compared to a non-TNBC patient to have high expression of at least one, or at least two, of the three RTKs *vs* fewer, adjusted for the above mentioned factors was 4.3 (95% CI 1.8–9.9, *P* = 0.001), and 5.3 (95% CI 2.0–13, *P* = 0.001) respectively.

### Gene copy number increase and comparison of high protein expression and increased gene copy number

FISH staining was evaluated in 193 (42%) patient tissue samples. The quality of the staining in the rest of the samples was too low to evaluate, or no staining was seen at all. A possible explanation for this is that we used tissue micro arrays of paraffin embedded tissue and a custom made quadruple probe, two factors known to complicate the FISH procedure [38].

**Figure 3:**
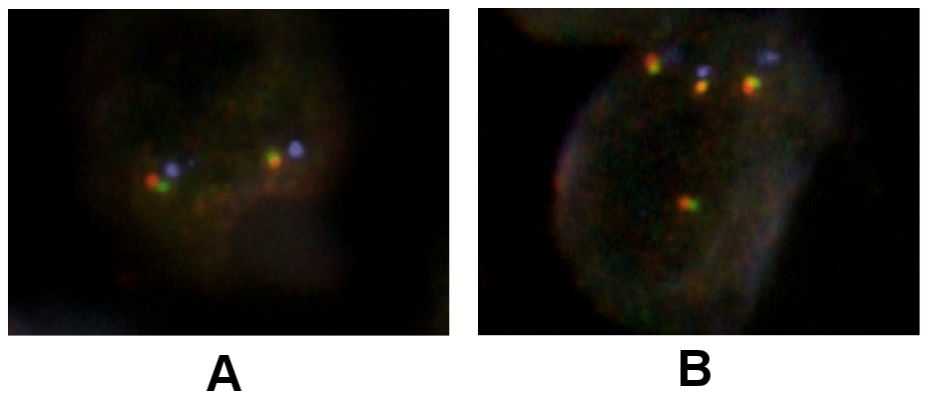
Examples of FISH. A shows a normal cell with two copies of each gene (green = *c-KIT*, yellow/gold = *VEGFR2* and red = *PDGFRα*), and two centromeres (blue). B shows a cell with a gene and centromere copy number gain. Original magnification ×63.

Examples of normal FISH staining pattern and positive FISH staining pattern are shown in Figure 3A and 3B. 21 (11%) of the 193 evaluated patient tumors were *c-KIT* FISH positive, 22 (11%) were *VEGFR2* FISH positive and 24 (12%) were *PDGFRα* FISH positive (Table 2). There was no difference in the percentage FISH positive tumors in the TNBC group compared to the non-TNBC group. No correlation was seen between the c-KIT, PDGFRα and VEGFR2 IHC positive tumors, and the respective marker FISH positive tumors (Table S3).

### Correlation between high protein expression, increased gene copy number and survival

**Figure 4:**
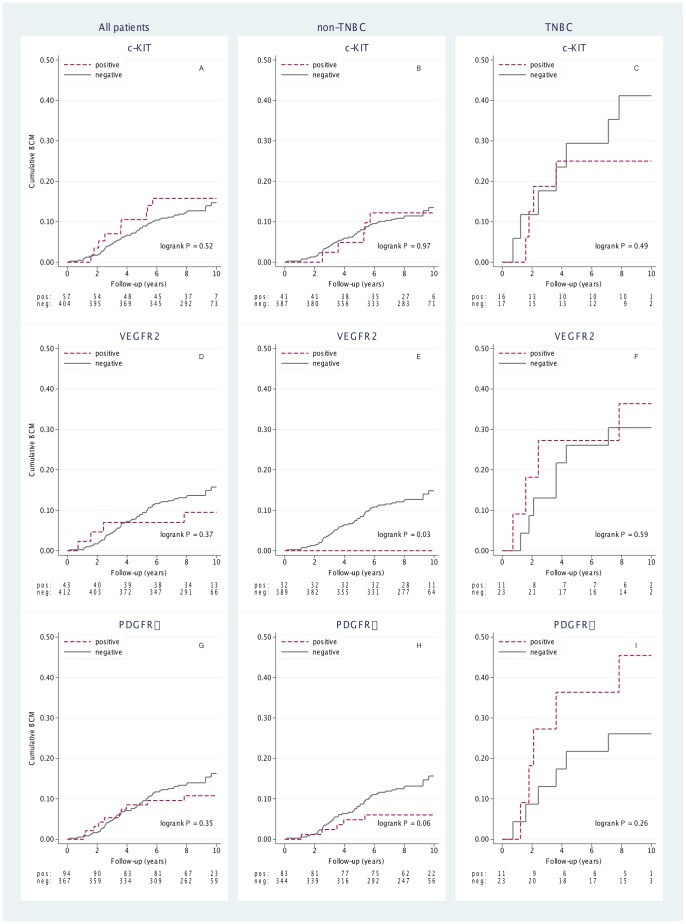
Breast cancer mortality (BCM) stratified for positive *vs* negative status of c-KIT (A, B and C), VEGFR2 (D, E and F) and PDGFRα (G, H and I) for all (A, D, G), non triple-negative (B, E, H) and triple-negative patients (C, F, I).

Cumulative incidence curves were calculated for patients with tumors having a high protein expression of c-KIT (Figure 4A, B and C), VEGFR2 (Figure 4D, E and F) and PDGFRα (Figure 4G, H and I) in both the non-TNBC (Figure 4B, E and H) and the TNBC group (Figure 4C, F and I). For c-KIT and PDGFRα, no statistically significant difference in BCM was seen for the IHC positive versus the IHC negative groups, neither for TNBC patients nor for non-TNBC patients. For VEGFR2 there was moderate evidence (*P* = 0.03) for lower BCM for the IHC positive patients in the non-TNBC group (Figure 4E), but no difference in the TNBC group (Figure 4F).

Cox regression showed no significant influence on mortality in the TNBC group for the tumors with high marker expression of c-KIT HR = 0.7 (95% CI 0.2–2.2), PDGFRα HR = 2.0 (95% CI 0.6–6.4), VEGFR2 HR = 1.4 (95% CI 0.4–4.8). In the non-TNBC group, a HR indicating notably lower breast cancer mortality was seen for the VEGFR2 positive patients, HR = 0.04 (95% CI 0.001–3.3), but this result was not significant (*P* = 0.16). For c-KIT and PDGFRα, the HRs showed no significant mortality influence in the non-TNBC group (c-KIT HR = 1.0, 95% CI 0.4–2.6 and PDGFRα HR = 0.4, 95% CI 0.2–1.0).

No difference in BCM was seen for FISH positive versus FISH negative patients, neither in the TNBC nor in the non-TNBC group (data not shown).

## Discussion

TNBC is a subgroup of breast cancer with poor prognosis and no targeted therapy available. In this study we show that TNBC is associated with high protein expression of the three RTKs and potential drug targets, c-KIT, VEGFR2 and PDGFRα, closely spaced at 4q12. The strongest evidence for correlation was seen for c-KIT and VEGFR2 while PDGFRα showed a somewhat weaker association. When RTK expression was adjusted for known tumor characteristics, the significant correlations for c-KIT and VEGFR2 expression were retained whereas the association between PDGFRα and TNBC was not significant. The low number of TNBC patients in the cohort can be one explanation for this. However, linking PDGFRα to TNBC is an interesting finding because PDGFRα has been detected as a key protein in one of the central epithelial to mesenchymal transition (EMT) processes, the invadopodia formation. EMT is a process where tumor cells lose epithelial characteristics and conversely gain invasive mesenchymal and stem cell-like features, and it has been related to breast cancer with a basal-like phenotype [39]. In invadopodia formation, PDGFRα is up-regulated and activated downstream of Twist1, and it is known that blocking of PDGFRα strongly decreases invadopodia formation [30].

Also, a recent study showed that mRNAs encoding PDGFRα, PDGFRβ and their ligand PDGF-C were highly expressed in basal B subtype of breast cancer cell lines with mesenchymal properties but not in luminal-like cell counterparts with more epithelial features [31]. High PDGFRα expression might thus be a sign of an active EMT process in the TNBC tumors.

Interestingly, Lehmann *et al.* have recently found that genes involved in the PDGFR and VEGF pathways are upregulated in the mesenchymal and mesenchymal stem-like TNBC subtypes [14]. It would thus be intriguing to investigate the expression of c-KIT, VEGFR2 and PDGFRα in different genomic TNBC subtypes. However the number of TNBC patients in the present cohort was too low for additional subdivision.

To further test the association between TNBC and the three RTKs encoded at 4q12, we analyzed how many tumors had a high expression of at least one, or at least two, of the three RTKs. We noticed a remarkably elevated frequency of high expression of either one of three markers (73.5% compared to 30.0%, *P*<0.001) or two of three markers (35.3% compared to 5.8%, *P*<0.001) in the TNBC compared to the non-TNBC. Also, the ORs for TNBC and high expression of at least one, or at least two, of the three RTKs were 4.3 and 5.3, respectively, when compared to non-TNBC, with *P*-values of 0.001. These results support a connection between TNBC and high expression of c-KIT, VEGFR2 and PDGFRα.

Survival analysis did not show any correlation between high protein expression of c-KIT, VEGFR2 or PDGFRα and changes in BCM in the TNBC subgroup. Previous studies have shown varying results; Nielsen *et al.*, 2004 reported no difference in survival for c-KIT positive BLBC patients compared to control [40], whereas Kashiwagi *et al.*, 2012 found a poorer outcome for c-KIT positive BLBC patients with a hazard ratio of 2.29 [26]. One explanation for these opposing results is differences in cut-point for categorizing a sample as positive; Nielsen *et al.*, 2004 used ≥25% stained cells as cut-point while Kashiwagi *et al.*, 2012 set their limit to ≥10%. In this study we used ≥1% stained cells as cut-point, since it is a standard cut-point for c-KIT assessment [25]. VEGFR2 has in one study been found to be significantly correlated to decreased breast cancer specific survival (BCSS) in TNBC patients [28]. In the present study, no such correlation was found. However, we found that VEGFR2 positive non-TNBC patients had a statistically significant lower mortality than VEGFR2 negative non-TNBC patients. A possible explanation for these differing results is that the previous study [28] included only premenopausal women with stage II breast cancer, while the present study had no upper age limit and a cohort weighted towards a low risk profile. Also, treatment regimens differed between the two studies. PDGFRα has been found to be associated with breast cancer progression and metastasis [29], [30]. In this study we did not find any association between poor survival and high PDGFRα expression.

193 (42%) of the 464 FISH stained tissue samples were assessable for gene copy number. No difference was seen in tissue samples with increased gene copy number between the TNBC and the non-TNBC group. Nor was any correlation seen between high protein expression and increased gene copy number. Survival analysis showed no correlation between increased gene copy number and differences in BCM.

*c-KIT*, *VEGFR2* and *PDGFRα* have previously been found to be amplified in 15–33% of primary glioblastomas [20], [41] and amplification of *c-KIT* and *PDGFRα* to be associated with poor survival of glioblastoma patients [21]. In a recent study, an increased copy number of the genes *c-KIT* and *VEGFR2* was found in the TNBC subgroup, and increased gene copy number was related to an aggressive phenotype and impaired prognosis [27]. These results were not confirmed in this study. Staining methods differed between the two studies where Johansson *et al.*, used fresh frozen tumor tissue, and we used formalin-fixed TMAs in the present study.

The results from this study suggest that a multi-targeting RTK inhibitor, such as for example sunitinib or sorafenib, would be a possible treatment option for TNBC patients. We found that as many as 73.5% of TNBC patients have a high expression of at least one of the three RTKs c-KIT, VEGFR2 and PDGFRα. A few pilot studies have been conducted evaluating sunitinib as treatment for metastatic breast cancer and in those studies TNBC patients showed promising response rates [42], [43]. However, a larger randomized phase III study on sunitinib as single treatment for metastatic breast cancer was aborted ahead of schedule since preliminary data showed a lower progression free survival amongst the sunitinib treated patients [44]. Important to notice is that the inclusion criteria in these studies were metastatic breast cancer regardless of subgroup or RTK expression (with the exception of the phase III randomized trial where HER2 positive patients were excluded). Since TNBC patients seemed to benefit from the anti-RTK treatment in pilot studies [42], [43], it is possible that only TNBC or BLBC patients should be included in a future study of anti-RTK treatment.

In addition to the RTK inhibitors described above, there are also drugs aimed at the RTK ligands such as bevacizumab targeting VEGF-A. In a recent study it was shown that addition of bevacizumab to TNBC patients in the neoadjuvant setting could increase the rate of pathologic complete response [45].

In conclusion, we have found that the proteins c-KIT, VEGFR2 and PDGFRα, encoded by genes at 4q12, are associated to the St Gallen breast cancer subgroup TNBC. No correlation was seen between high protein expression, increased gene copy number and BCM in the TNBC group. A remarkably high expression of at least one, and at least two, of the three investigated markers was seen in the majority of TNBC patients compared to non-TNBC, which suggests that anti-RTK therapy could be useful in this patient group in the future.

## Supporting Information

Table S1 **Presents correlations between c-KIT, VEGFR2 and PDGFRα.**
(DOCX)

Table S2 **A, b and c presents the distribution of the tumors between the percentage groups (c-KIT) and the IHC scores (VEGFR2 and PDGFRα) for TNBC and non-TNBC.**
(DOCX)

Table S3 **Shows how IHC and FISH results relate.**
(DOCX)
