# Supplementary material for: The Three Receptor Tyrosine Kinases c-KIT, VEGFR2 and PDGFRα, Closely Spaced at 4q12, Show Increased Protein Expression in Triple-Negative Breast Cancer
Source: PLoS One. 2014 Jul 15;9(7):e102176. doi: 10.1371/journal.pone.0102176 (PMC4098911; doi:10.1371/journal.pone.0102176)
Supplement: Table S1 — Presents correlations between c-KIT, VEGFR2 and PDGFRα. (DOCX) [file pone.0102176.s001.docx]

**Table S1.** Correlations between c-KIT, VEGFR2 and PDGFRα.

|  |  | **c-KIT** | **VEGFR2** | **PDGFRα** |
| --- | --- | --- | --- | --- |
| **c-KIT** | Pearson Correlation | 1 |  |  |
|  | Sig. (2-tailed) |  |  |  |
|  | N | 461 |  |  |
| **VEGFR2** | Pearson Correlation | 0.06 | 1 |  |
|  | Sig. (2-tailed) | 0.21 |  |  |
|  | N | 452 | 455 |  |
| **PDGFRα** | Pearson Correlation | 0.07 | 0.19 | 1 |
|  | Sig. (2-tailed) | 0.12 | <0.001 |  |
|  | N | 458 | 453 | 461 |
