# Supplementary material for: The Three Receptor Tyrosine Kinases c-KIT, VEGFR2 and PDGFRα, Closely Spaced at 4q12, Show Increased Protein Expression in Triple-Negative Breast Cancer
Source: PLoS One. 2014 Jul 15;9(7):e102176. doi: 10.1371/journal.pone.0102176 (PMC4098911; doi:10.1371/journal.pone.0102176)
Supplement: Table S2 — A, b and c presents the distribution of the tumors between the percentage groups (c-KIT) and the IHC scores (VEGFR2 and PDGFRα) for TNBC and non-TNBC. (DOCX) [file pone.0102176.s002.docx]

**Table S2a.** Percentage of c-KIT expression in TNBC *vs* non-TNBC.

| **c-KIT % positive cells** | **All patients N (%)** | **non-TNBC N (%)** | **TNBC N (%)** |
| --- | --- | --- | --- |
|  | N=461 | N=428 | N=33 |
| 0<1 | 404 (87.6) | 387 (90.0) | 17 (51.5) |
|  |  |  |  |
| ≥1<5^a^ | 2 (0.4) | 2 (0.5) | 0 |
| ≥5<10 | 7 (1.5) | 6 (1.4) | 1 (3.0) |
| ≥10<20 | 5 (1.1) | 5 (1.2) | 0 |
| ≥20<30 | 6 (1.3) | 5 (1.2) | 1 (3.0) |
| ≥30<40 | 2 (0.4) | 1 (0.2) | 1 (3.0) |
| ≥40<50 | 3 (0.7) | 2 (0.5) | 1 (3.0) |
| ≥50<60 | 0 | 0 | 0 |
| ≥60<70 | 4 (0.9) | 3 (0.7) | 1 (3.0) |
| ≥70<80 | 4 (0.9) | 3 (0.7) | 1 (3.0) |
| ≥80<90 | 5 (1.1) | 4 (0.9) | 1 (3.0) |
| ≥90<95 | 11 (2.4) | 7 (1.6) | 4 (12.1) |
| ≥95<100 | 2 (0.4) | 1 (0.2) | 1 (3.0) |
| =100 | 6 (1.3) | 2 (0.5) | 4 (12.1) |

^a^A tumor sample was considered c-KIT positive whenever the percentage stained cells was ≥1.

**Table S2b.** Scores for VEGFR-2 expression in TNBC *vs* non-TNBC.

| **VEGFR-2 score** | **All patients N (%)** | **non-TNBC N (%)** | **TNBC N (%)** |
| --- | --- | --- | --- |
|  | N=455 | N=421 | N=34 |
| 0 | 68 (14.9) | 66 (15.7) | 2 (5.9) |
| 1 | 6 (1.3) | 6 (1.4) | 0 |
| 2 | 26 (5.6) | 24 (5.6) | 2 (5.9) |
| 3 | 207 (44.6) | 193 (44.9) | 14 (41.2) |
| 4 | 5 (1.1) | 5 (1.2) | 0 |
| 6 | 100 (21.6) | 95 (22.1) | 5 (14.7) |
|  |  |  |  |
| 9^b^ | 43 (9.5) | 32 (7.4) | 11 (32.4) |

^b^A tumor sample was considered VEGFR2 positive whenever the histoscore was >6.

**Table S2c.** Scores for PDGFR-α expression in TNBC *vs* non-TNBC.

| **PDGFR-α score** | **All patients N (%)** | **non-TNBC N (%)** | **TNBC N (%)** |
| --- | --- | --- | --- |
|  | N=461 | N=427 | N=34 |
| 0 | 240 (52.1) | 223 (52.2) | 17 (50.0) |
| 1 | 2 (0.4) | 2 (0.5) | 0 |
| 2 | 13 (2.8) | 12 (2.8) | 1 (2.9) |
| 3 | 26 (5.6) | 26 (6.1) | 0 |
| 4 | 86 (18.5) | 81 (19.0) | 5 (14.7) |
|  |  |  |  |
| 6^c^ | 3 (0.6) | 2 (0.5) | 1 (2.9) |
| 8 | 60 (12.9) | 54 (12.6) | 6 (17.6) |
| 9 | 0 | 0 | 0 |
| 12 | 31 (6.7) | 27 (6.3) | 4 (11.8) |

^c^A tumor sample was considered PDGFRα positive whenever the histoscore was ≥5.
