# Supplementary material for: The Three Receptor Tyrosine Kinases c-KIT, VEGFR2 and PDGFRα, Closely Spaced at 4q12, Show Increased Protein Expression in Triple-Negative Breast Cancer
Source: PLoS One. 2014 Jul 15;9(7):e102176. doi: 10.1371/journal.pone.0102176 (PMC4098911; doi:10.1371/journal.pone.0102176)
Supplement: Table S3 — Shows how IHC and FISH results relate. (DOCX) [file pone.0102176.s003.docx]

**Table S3.** Relationship between immunohistochemical (IHC) and fluorescence *in situ* hybridisation (FISH) results.

|  | **Total number of patients with FISH results available N (%)** | **FISH negative for respective marker N (%)** | **FISH positive for respective marker N (%)** | ***P*-value** |
| --- | --- | --- | --- | --- |
| **c-KIT** |  |  |  |  |
| **IHC positive** | 30 (100) | 26 (87) | 4 (13) | 0.75^a^ |
| **IHC negative** | 172 (100) | 154 (90) | 18 (11) |  |
| **VEGFR2** |  |  |  |  |
| **IHC positive** | 9 (100) | 9 (100) | 0 | 0.6^a^ |
| **IHC negative** | 193 (100) | 170 (88) | 23 (12) |  |
| **PDGFRα** |  |  |  |  |
| **IHC positive** | 17 (100) | 16 (94) | 1 (6) | 0.7^a^ |
| **IHC negative** | 174 (100) | 151 (87) | 23 (13) |  |

^a^P-value from Fisher’s Exact Test.
